# Prediction of the Vaccine-derived Poliovirus Outbreak Incidence: A Hybrid Machine Learning Approach

**DOI:** 10.1038/s41598-020-61853-y

**Published:** 2020-03-19

**Authors:** Ahmed A. Hemedan, Mohamed Abd Elaziz, Pengcheng Jiao, Amir H. Alavi, Mahmoud Bahgat, Marek Ostaszewski, Reinhard Schneider, Haneen A. Ghazy, Ahmed A. Ewees, Songfeng Lu

**Affiliations:** 1 Luxembourg Centre for Systems Biomedicine (LCSB), University of Luxembourg, EschsurAlzette, Luxembourg; 2 Department of Mathematics, Faculty of Science, Zagazig University, Zagazig, Egypt; 3 Ocean College, Zhejiang University, Zhoushan, 316021 Zhejiang China; 4 Department of Civil and Environmental Engineering, University of Pittsburgh, Pittsburgh, PA USA; 5 Department of Bioengineering, University of Pittsburgh, Pittsburgh, PA USA; 6 Research Group Immune- and Bio-markers for Infection, the Center of Excellence for Advanced Sciences, the National Research Center, Cairo, Egypt; 7 Therapeutic Chemistry Department, the National Research Center, Cairo, Egypt; 8 Biotechnology department, Animal Health research institute, Kafrelsheikh, Egypt; 9 Department of Computer, Damietta University, Damietta El-Gadeeda City, Egypt; 10 School of Computer Science& Technology, Huazhong university of Science and Technology, Wuhan, 430074 China; 11 Department of Computer Science and Information Engineering, Asia University, Taichung, Taiwan

**Keywords:** Computational models, Data mining

## Abstract

Recently, significant attention has been devoted to vaccine-derived poliovirus (VDPV) surveillance due to its severe consequences. Prediction of the outbreak incidence of VDPF requires an accurate analysis of the alarming data. The overarching aim to this study is to develop a novel hybrid machine learning approach to identify the key parameters that dominate the outbreak incidence of VDPV. The proposed method is based on the integration of random vector functional link (RVFL) networks with a robust optimization algorithm called whale optimization algorithm (WOA). WOA is applied to improve the accuracy of the RVFL network by finding the suitable parameter configurations for the algorithm. The classification performance of the WOA-RVFL method is successfully validated using a number of datasets from the UCI machine learning repository. Thereafter, the method is implemented to track the VDPV outbreak incidences recently occurred in several provinces in Lao People’s Democratic Republic. The results demonstrate the accuracy and efficiency of the WOA-RVFL algorithm in detecting the VDPV outbreak incidences, as well as its superior performance to the traditional RVFL method.

## Introduction

Poliovirus (PV) surveillance is considered as one of the most challenging issues in countries with suboptimal vaccination coverage levels due to the repetitive silent circulation of the vaccine derived poliovirus (VDPV). Despite its durable intestinal and humoral immunity, VDPV is genetically instable that might revert to wild-type virulence. According to a number of studies^1^, vaccines can cause vaccine-associated flaccid paralysis. Noteworthy, it can replicate for a prolonged time coinciding with the suboptimal vaccination^1^. Interaction of PV and CD155 receptors facilitates its entry^2^. Thereafter, the viral RNA is released. The genome enclosed in the viral particle is used as mRNA and translated by the host cell. The virus hijacks the cell’s translation, leading to inhibition of protein synthesis during viral protein production. Ribosome entry site directs the viral RNA translation and synthesis of (+) RNA occurs. Some of the (+) RNA are used as templates for (−) RNA synthesis, some function as mRNA, and some are destined to be the genomes of virus progeny^1^.

Globally, the concerted surveillance with the continuous integration and interpretation of health-related data are required to keep the prevention and elimination programs updated. PV surveillance is considered as one of the most important element of the Global Polio Eradication Initiative (GPEI) endgame strategy, which is also useful in detecting VDPV. There are mainly two types of PV surveillance, including (1) the environmental surveillance (ES) that analyzes wastewater to detect if the current collected samples carry PV^3,4^, and (2) the acute flaccid paralysis surveillance (AFPS) that depends on clinical presentation. However, the traditional PV surveillance methods are resource intensive to maintain the system of AFPS for the long term^2^. Still, combatting disease outbreaks significantly depends on gathering data from clinicians or laboratories and developing associated central information repositories. These are usually inefficient processes that might lead to further spread of disease^5–9^. Consequently, the important and yet to be solved issue related to PV surveillance is *how to* rapidly unveil outbreak incidences. A powerful solution to deal with this issue is machine learning (ML). ML has been increasingly utilized for solving complex real-world problems, its application in public health arguably needs more attention. In this context, the ML methods have been successfully applied to in public health problems such as the real-time detection of foodborne illness^10^, and syndromic surveillance that depends on the reporting symptoms of the patients^11,12^. Tessmer *et al*.^13^ proposed various ML techniques such as artificial neural networks (ANN), convolutional neural network (CNN), and long-short term memory (LSTM) to determine the parameter of basic reproduction number. These methods were applied to epidemiological data from outbreaks of influenza A(H1N1) pdm09, mumps, and measles. Moreover, the ML methods are used for syndromic surveillance based on chief complaint field to detect disease outbreaks. For example, Lee *et al*.^14^ compared two recurrent ANN models based on LSTM and gated recurrent unit (GRU) cells, multinomial naive Bayes (MNB) and support vector machine (SVM) to improve the syndromic surveillance. Volkova *et al*.^15^ utilized ANNs to forecast the influenza-like illness dynamics for military populations. To the best of our knowledge, however, most of the machine learning prediction models in public health are based on ANNs and their extensions (e.g.^16,17^). Although the traditional ANN method is a powerful method for classification, clustering, and regression^18,19^, certain limitations are reported due to its basic structures, namely, the trapping in local minima and initialization process that involves assigning initial random values to the weights of the network^20^. Those limitations severely impede the applications of ANN-based methods in public health.

To overcome the critical issues in ANN, random vector functional link network (RVFL) has been developed as a single feed-forward neural networks based on a randomized algorithm^21,22^. Thanks to the growing concept of randomization, the RVFL method considers the link between inputs and outputs and therefore, effectively overcomes the limitations of traditional ANN algorithms. On this basis, the weights connecting the input and hidden layers are randomly generated and then fixed during the updating phase using Moore-Penrose pseudo-inverse theory^23^. RVFL has also been reported with other features, e.g., fast convergence^24^, good approximation capability^22^, and compatibility for real-time applications with simple implementation of hardware^20^. Given its unique characteristics, RVFL has been used in several applications including remote sensing^25^, big data analytics^26^, forecasting temperature distribution^27^, short-term electricity load demand forecasting^28^, time-series data prediction^29^, language handwritten script recognition^30^, and semi-supervised learning^31^. However, the efficiency of RVFL is significantly affected by its parameters. Studies have been conducted to determine the influence of parameters on the RVFL’s efficiency. Park *et al*.^17^ concluded that a significant effect was found on the performance of RVFL when direct links were used between input and output layers. Additionally, the Radbas function provided RVFL with higher ability of reaching targets compared to using sign or hardlim as activation function^17^. Li *et al*.^32^ investigated the relation between the domain of hidden parameters and the performance of RVFL and found that it was not suitable to generate hidden weights from fixed domain such as [−1,1]^32^. Zhang and Suganthan^33^ conducted a comprehensive study to find the best parameters that enhance the performance of RVFL. In the same manner to traditional ANN, the process of randomly selecting RVFL network parameters typically leads to high complexity. Taking the advantages of the swarm optimization algorithm that emulates the social behavior of the whales to attack their prey^34^, whale optimization algorithm (WOA) offers a powerful tool to address the problem of finding suitable configuration in RFVL.

The classes include the IgG antibodies in Children (n = 1216) and adults (n = 1228), including health care workers and blood donors. Antibody titers in a subset of classes resulted from microneutralization show 92% of children class had anti-poliovirus antibodies. On the other hand, the antibodies seroprevalences were 81.7% and 71.9% in adult blood donors and healthcare worker, respectively. Noteworthy, both children and adult classes show the neutralizing antibodies against one of the three poliovirus serotypes and had antibodies against all serotypes. These findings were compatible with the epidemiology of the outbreak [41].

The classification supports the medical field to optimize the evaluation of the vaccination schemes in diverse cohorts using the seroprevalence of poliovirus antibodies. Additionally, to sustain the value of an ELISA in the developed countries with specific epidemiological nature. To date, acceptable underestimation of vaccine scheme in children by ELISA resulted; however, the low sensitivity of the ELISA in the adults. Thus, the classification paradigm supports ELISA to be a reasonable alternative to the microneutralization in children classes. Using classification model by countries with uncertain vaccination schemes and limited resources, enable them not only to avoid the risk of outbreaks from poliovirus vaccines but also to prevent the re-importation of wild strains moreover, this will improve ELISA for classes studies to judge the immunization programs.

In this study, we develop a hybrid ML paradigm by implementing WOA in RVFL to accurately track the immunity response of VDPV during the outbreaks. In the hybrid WOA-RVFL method, the domain search for the parameters in RVFL (i.e., number of neurons, activation function, link between input and output) is first determined. Thereafter, a random population is generated in which each solution represents a configuration of the RVFL network. The solutions of the population are updated using the best solutions and the operators of the WOA. The process of updating the solution is repeated until the best configuration is obtained. The results show that the presented hybrid approach lead to improving the performance of the RVFL algorithm for the prediction of the VDPV outbreak incidences.

## Methods

In this section, basic details about the RVFL and WOA are briefly described followed by the description of the proposed hybrid WOA-RVFL method.

### Random vector functional link networks

**Figure 1:**
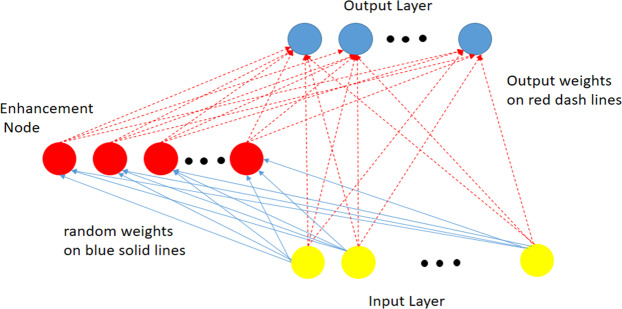
Structure of the RVFL network.

RVFL benefits from the properties of random weights and the functional link^27^. In general, the RVFL algorithm has the same structure as the single layer feedforward neural network (SLFNN) except for a direct connection between the input and output neurons. This type of connection improves the ability of RVFL to avoid overfitting. Figure 1 shows the structure of the RVFL network. It can be seen that where the neuron at the input layer receives the dataset $Y=\{({y}_{i},\,{z}_{i})|{y}_{i}\in {R}^{n},{z}_{i}\in {R}^{m},i=1,\ldots ,N,$ then each hidden neuron (enhancement) computes its output by:

1 $${O}_{j}({a}_{j}{y}_{i}+{b}_{j})=\frac{1}{1+{e}^{-({a}_{j}{y}_{i}+{b}_{j})}},{a}_{j}\in [-S,S],{b}_{j}\in [0,S],\,\,j=1,2,\ldots ,{N}_{h}$$

where *b*_*j*_ and *a*_*j*_ are the bias and the weight between the input and enhancement neurons, respectively. $S$ represents a scale factor updated during the learning process for each dataset. The output of RVFL is computed using the output weight ($w\in {R}^{n+P})$ defined as:

2 $$Z=Bw$$

where *B* represents the input matrix to the output layer (i.e., the input data and the output of the enhancement neurons), and it is defined as:

3 $$B=[{B}_{1}{B}_{2}]$$

$${B}_{1}=[\begin{array}{ccc}{y}_{11} & \ldots  & {y}_{1n}\\ \vdots  & \ddots  & \vdots \\ {y}_{N1} & \ldots  & {y}_{Nn}\end{array}],{B}_{2}=[\begin{array}{ccc}{O}_{1}({a}_{1}{y}_{1}+{b}_{1}) & \ldots  & {O}_{P}({a}_{P}{y}_{1}+{b}_{P})\\ \vdots  & \ddots  & \vdots \\ {O}_{1}({a}_{1}{y}_{N}+{b}_{1}) & \ldots  & {O}_{P}({a}_{P}{y}_{N}+{b}_{P})\end{array}]$$

In order to update $w$ in Eq. (2), Moore-Penrose pseudo-inverse or the ridge regression^27^ can be used as defined, respectively:

4 $$w={B}^{\dagger }Z$$

and

5 $$w={\left(\frac{I}{C}+{B}^{T}B\right)}^{-1}{B}^{T}Z,$$

where $I$ and *C* are the identity matrix and trading-off parameter, respectively. Note that $\dagger$ is the Moore-Penrose pseudo-inverse.

### Whale optimization algorithm

WOA was proposed as a swarm algorithm to simulate the behaviors of whales during the process of attacking the prey^34^. This process can be described by two approaches, including (1) encircling and (2) bubble-net.

In the encircling approach, each whale (${x}_{i},\,i=1,2,\ldots ,N$) updates its location at current iteration ($t$) based on the distance (${D}_{i}$) to the prey (${x}^{\ast }$) as:

6 $${x}_{i}(t+1)={x}^{\ast }(t)-A\odot {D}_{i},{D}_{i}=|B\odot {x}^{\ast }(t)-x(t)|$$

where $\odot$ is the element-wise multiplication, and the two coefficients $\,A$ and $b$ are updated as

7 $$A=2a\odot r-a,\,{\rm{and}}\,B=2r.\,$$

In Eq. (7), the parameter *a* is decreased from 2 to 0 with the increasing of the iterations (i.e., $a=a-\frac{ta}{{t}_{max}}$, where ${t}_{max}$ represents the maximum number of iterations). The value of $r$ is randomly generated in [0,1] interval.

In the bubble-net method, the location of the whale ${x}_{i}$ is updated using spiral, which simulates the movement of ${x}_{i}$ around ${x}^{\ast }$ using the helix-shaped^34^ as:

8 $${x}_{i}(t+1)={x}^{\ast }(t)+{D}_{i}\odot {e}^{bl}\odot \,\cos (2\pi l),$$

where $b$ is a random number, $l$ is a parameter determine the shape of a logarithmic spiral. The whales can swim around the prey simultaneously using the spiral-shaped path and shrinking circle based on the probability $p\in [0,1]$ as follows:

9 $${x}_{i}(t+1)=\{\begin{array}{ll}{x}^{\ast }(t)-A\odot {D}_{i}, & \,if\,p\ge 0.5\,\\ {x}^{\ast }(t)+{D}_{i}\odot {e}^{bl}\odot \,\cos (2\pi l) & \,otherwise\end{array}$$

In addition, it is possible to update the location of each whale based on the location of the random whale ${x}_{r}$ as:

10 $${x}_{i}(t+1)={x}_{r}(t)-A\odot {D}_{r},\,\,{D}_{r}=|B\odot {x}_{r}(t)-{x}_{i}(t)|$$

The final steps of the traditional WOA can be summarized in Algorithm 1.

**Algorithm 1:**
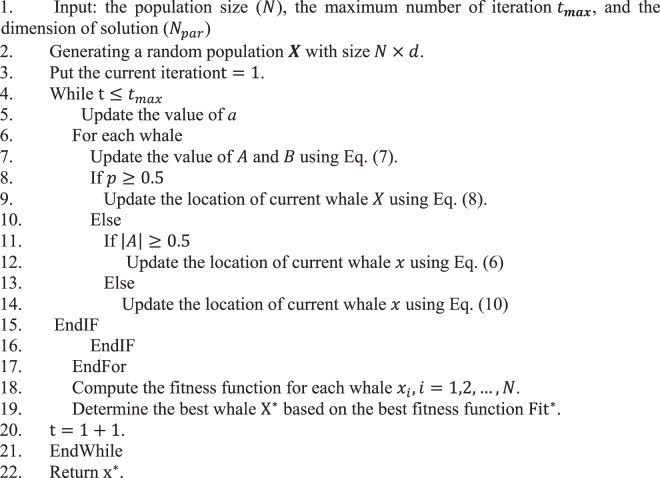
The Whale Optimization Algorithm.

### The proposed WOA-RVFL method

The proposed method for classification of the VDPV outbreak incidence is based on the integration of the RVFL and WOA algorithms. In WOA-RVFL, WOA is used to find the best configuration of the parameters for the RVFL network. The proposed WOA-RVFL approach consists of two stages: (1) learning stage and (2) evaluating stage. In the learning stage, WOA-RVFL starts with splitting the dataset into training, validation and testing sets, and then generating a random population $X$ with $N$ solutions. Each solution represents one configuration for the RVFL network. Thereafter, RVFL is constructed based on the parameters inside the current solution. The RVFL network is trained using the training set and then validated using the validation set. After evaluating all solutions within population $X$, the best solution is determined. The population $X$ is then updated by the operators of WOA. These steps are repeated until the termination criteria are met. Meanwhile, the second stage starts with constructing the RVFL network using the best configuration, and then evaluating it the network using the testing data.

### Learning stage

In this stage, the dataset is divided into three sets: training, validation and testing. The training and validation sets are used during this stage. The next step is to generate a population $X$ that contains ${N}_{cf}$ and each solution has dimension ${N}_{par}$ as:

11 $${x}_{ij}={l}_{j}+rand\times ({u}_{j}-{l}_{j}),i=1,\ldots ,{N}_{con},j=1,\ldots ,{N}_{par}$$

where ${u}_{j}$ and ${l}_{j}$ represent the upper and lower boundary of the *j*^th^ parameter, respectively. In order to explain this process, consider that the current solution is ${x}_{i}=[{x}_{i1},{x}_{i2},{x}_{i3},{x}_{i4},{x}_{i5},{x}_{i6},{x}_{i7}]=[{N}_{h},Bias,link,AF,RT,mode,Scal{e}_{m}]$. ${N}_{h}$ is the number of hidden neurons; $Bias$ is the parameter that determines if there is a bias in the output neurons; $link$ refers to the network direct link to output layer; $AF$ is the Activation Function (hardlim, sign, sig, radbas, sin, and tribas); $RT$ represents the type of randomization methods used to generate the weights here (Uniform, and Gaussian); $mode$ represents the method used to update the weights (regularized least square, and Moore-Penrose pseudoinverse); and $Scal{e}_{m}$ is a parameter representing the scaling the features (i.e., scale the feature for 1) all neurons, 2) each hidden neuron separately, and 3) the range of the randomization for uniform distribution. For instance, ${x}_{i}=[200,1,1,3,1,2,1]$ means that the number of neurons is 200 and there are bias and direct link. The other numbers (3,1,2,1) indicate that the sig function, Uniform, Moore-Penrose pseudoinverse, scale the feature for all neurons are used, respectively.

The next step is to construct the RVFL network using the current solution ${x}_{i}$, using the training set to train the current RVFL, and using the validation set to evaluate the trained network and compute the error between the prediction value and original value of the target using the following equation:

12 $$Fit=1-\theta $$

where $\theta$ represents the accuracy of the current RVFL network. Thereafter, the best solution is selected and the current population $X$ is updated using the steps of the WOA as discussed in Algorithm 1. The process of updating the solutions of $X$ is repeated until the termination criteria are met.

### Evaluation stage

**Figure 2:**
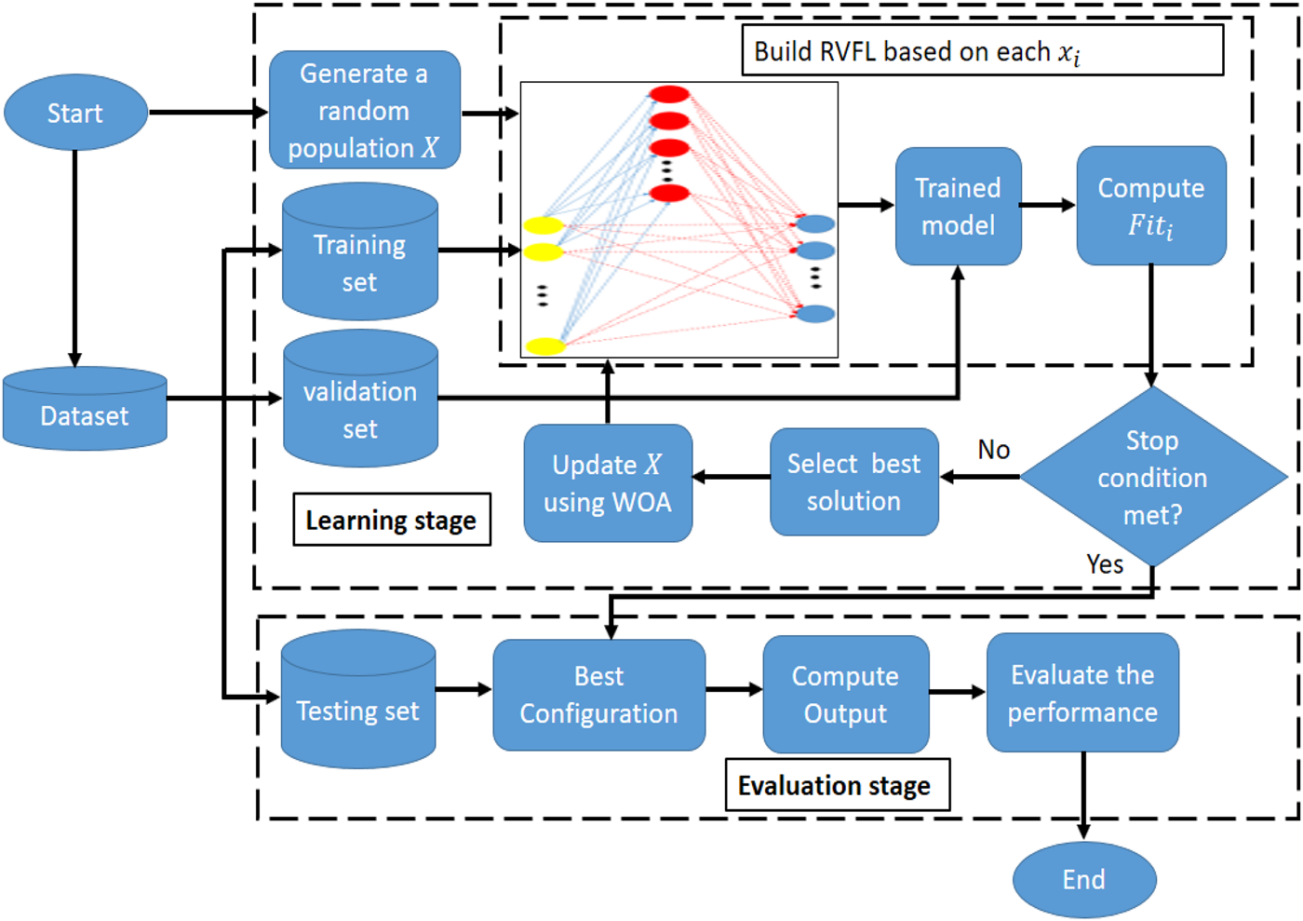
The WOA-RVFL classification process.

This stage starts with selecting the best configuration of RVFL and evaluating its accuracy on the testing data using different performance measures. The WOA-RVFL classification process is illustrated in Fig. 2.

### Experimental study

The experimental study is conducted in two phases. The WOA-RVFL algorithm is first benchmarked using 11 UCI machine learning datasets [40]. Thereafter, the method is implemented for the prediction of the VDPV outbreak incidences. In order to analyze the performance of the WOA-RVFL method, a set of performance measures is used, including the Accuracy, Precision, and Recall as

Accuracy:

13 $$Acc=\frac{TP+TN}{TP+TN+FP+FN}$$

Precision:

14 $$Pre=\frac{TP}{TP+FP}$$

and Recall:

15 $$Rec=\frac{TP}{TP+FN}$$

where $TP$, $TN$, $FP$, and $FN$ denotes the true positive, true negative, false positive, and false negative samples, respectively.

### Phase I: UCI Datasets

The performance of the proposed method is evaluated using the widely-used set of UCI datasets given in Table 1. The datasets have different characteristics which makes their classification a challenging problem. For each case, the available datasets are randomly divided into training (80%), validation (10%) and testing (10%) subsets.

**Table 1:** The UCI datasets.

NO	Dataset	Features	Sample	No of class	Subject
1	Clean 1	168	476	2	Physical
2	Clean 2	168	6598	2	Physical
3	Hayes-roth	5	160	3	Social
4	IonoSphere	34	351	2	Physical
5	House-votes	16	435	2	Social
6	Madelon	500	4400	2	N/A
7	PCMAC	3289	1943	2	N/A
8	Soybean	35	307	19	Life
9	WaveForme	40	5000	3	Physical
10	Wine	13	178	3	Physical
11	Zoo	17	101	7	Life

## Results and discussion

**Figure 3:**
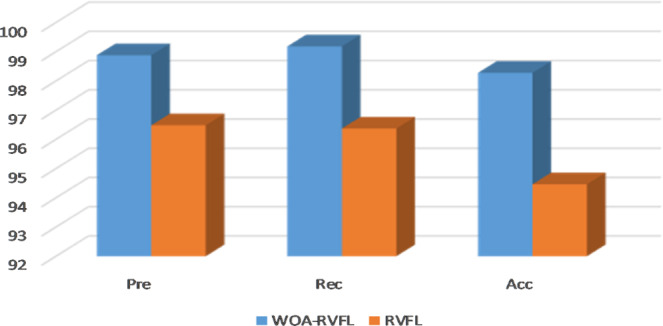
Accuracy, Precision and Recall rates of the WOA-RVFL and RVFL methods for the training set (UCI data).

**Figure 4:**
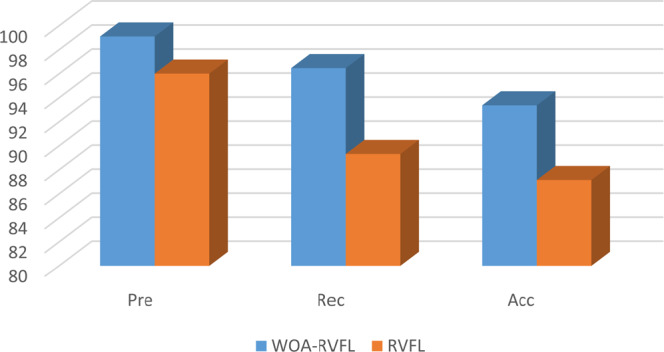
Accuracy, Precision and Recall rates of the WOA-RVFL and RVFL methods for the validation set (UCI data).

**Figure 5:**
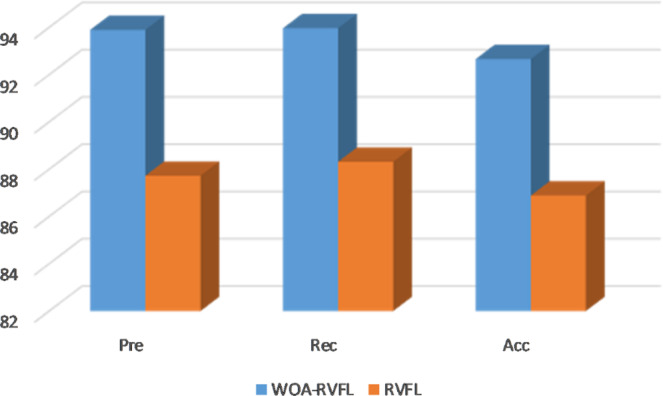
Accuracy, Precision, and Recall of the WOA-RVFL and RVFL methods for the testing set (UCI data).

The results of a comparative study between the proposed WOA-RVFL method and the traditional RVFL algorithm are shown Table 2 and Figs. 3–5. The parameter settings that provide the best predictions are as follows: For the WOA algorithm, parameter *a* was set to 2, and *b* = 1. Also, the optimal size of population and the total number of iterations were 20. The parameters of the traditional RVFL algorithm were set based on some recommended values^23^ and after a trial and error approach. Accordingly, radbas was taken as the activation function ($AF$), with a Bias and a link between the input and output (i.e. Bias = 1 and link = 1). The ridge regression was used to update the weights (i.e., mode = Ridge Regression). The optimal number of hidden neurons was 200, and Scale_m_ = 1. Both of the algorithms were implemented in Matlab 2017b in Windows 10 64-bit environment using a PC with 4 G RAM and an Intel® Core™ i3-3110M Processor. On average, the CPU times for the training of the WOA-RVFL and RVFL algorithms were, respectively, 0.3936 s and 0.3833 s. As seen in Table 2, the performance of the proposed WOA-RVFL is notably better than RVFL in nearly all cases. The Precision, Accuracy and Recall rates of the proposed WOA-RVFL method are higher than RVFL on the training, validation and testing data. This clearly indicates that introducing the WOA into the RVFL algorithm has improved both its learning and generalization capabilities. This superior performance is more noticeable for six datasets (Zoo, Wine, PCMAC1, Hayseroth, HouseVote, Madelon).

**Table 2:** Performance statistics of the WOA-RVFL and RVFL methods over different UCI datasets.

Dataset	Set	WOA-RVFL			RVFL		
		Pre	Rec	Acc	Pre	Rec	Acc
Zoo	Train	100	100	100	100	100	100
	Validation	100	100	77.80	100	100	66.76
	Test	85.71	85.71	100	71.43	64.29	90.00
Wine	Train	100	100	100	97.47	97.71	97.52
	Validation	100	100	100	97.24	95.91	100
	Test	100	100	100	100	100	100
Soybean	Train	100	100	100	100	100	100
	Validation	100	100	100	100	100	100
	Test	100	100	100	100	100	100
PCMAC1	Train	100	100	100	100	100	100
	Validation	95.36	96.87	100	91.23	89.79	92.70
	Test	91.21	91.36	91.24	87.63	87.63	87.63
Madelon	Train	100	100	100	74.32	74.32	74.32
	Validation	60.48	67.81	61.32	48.87	46.16	43.59
	Test	68.46	68.47	68.46	55.00	55.00	55.00
Ionosphere	Train	100	100	100	100	100	98.10
	Validation	100	100	100	99.29	100	100
	Test	100	100	95.71	100	100	94.29
House-Vote	Train	100	100	100	97.47	96.74	97.19
	Validation	94.87	100	91.67	94.87	97.79	92.23
	Test	94.19	93.70	94.25	93.17	92.19	93.02
Hayesroth	Train	87.68	91.01	85.71	92.03	91.31	90.76
	Validation	94.67	100	91.69	47.61	63.52	61.53
	Test	93.33	94.44	92.31	57.78	72.38	61.54
Clean2	Train	100	100	100	100	100	95.23
	Validation	100	100	100	100	100	97.25
	Test	100	100	100	100	100	94.39
Clean1	Train	100	100	100	100	100	95.12
	Validation	100	100	100	100	100	100
	Test	100	100	100	100	100	95.30

Moreover, from Figs. 3–5 it can be noticed that the high performance of the proposed WOA-RVFL against the traditional RVFL in terms of Precision, Accuracy and Recall. By analysis the behaviors of the WOA-RVFL during the training phase, it can be observed that the difference between the accuracy, recall, and precision of the WOA-RVFL and the traditional RVFL is nearly 3%, 4%, 2.5%, respectively. Whereas, during the validation phase the difference between them in

terms of accuracy, recall, and precision is 6%, 7%, 5%, respectively. Also, by observed the difference between the proposed WOA-RVFL and the traditional RVFL by using the testing set it can be found it is nearly, the same of performance during validation phase, 6%, 5%, and 4%, for accuracy, recall, and precision, respectively.

Moreover, the Friedman (FD) test is used to determine if there is a significant difference between the WOA-RVFL and traditional RVFL. The results of FD are given in Table 3, it can be noticed that the proposed has mean rank better than the traditional RVFL according to the precision, recall, and accuracy among all the tested dataset and the partitions of the datasets (i.e., the row with name average). In addition, there is a significant difference between the WOA-RVFL and RVFL. However, by comparing the results over the training, Validation, and testing set, it can be noticed that there is no significant difference, but the proposed WOA-RVFL has the best mean rank overall these sets.

**Table 3:** The mean rank and p-value of Friedman test to compare between WOA-RVFL and RVFL.

		Pre		Rec		Acc	
		WOA-RVFL	RVFL	WOA-RVFL	RVFL	WOA-RVFL	RVFL
Training set	Mean Rank	1.6000	1.4000	1.5500	1.4500	1.7500	1.2500
	p-value	0.3173		0.5637		0. 0588	
Validation	Mean Rank	2.8000	2.2000	2.9000	2.10	2.8000	2.2
	p-value	0.2076		0.0881		0.2453	
Test	Mean Rank	1.75	1.25	2.8500	2.15	3.0500	1.950
	p-value	0.0253		0.1587		0. 0423	
Average	Mean Rank	1.7	1.3	2.8167	2.1833	2.9333	2.0667
	p-value	0.0013		0.0068		0.0025	

### Phase II: Prediction of the VDPV outbreak incidences

In this section, the WOA-RVFL algorithm is trained, validated and tested using available data related to a recent VDPV outbreak occurred in several provinces in Lao People’s Democratic Republic (Lao PDR) [41].

The database includes serum samples from different urban cohorts collected before poliomyelitis outbreak in Lao PDR in 2015. Data was approved by the National Ethics Committee for Health Research of the Ministry of Health in the investigated area (Reference: NECHR 2013-860, 2013-732, 2014-059, 2013-038 and 2017-016). The National Ethics Committee for Health Research of the Ministry of Health in Lao (Ethical approval reference NECHR 2013–860, 2013–732, 2014–059, 2013–038 and 2017–016) approved the open data with the Creative Commons Attribution 4.0 license. More details about this database can be found in [41].

The cohorts included in this study are given as follows:

Fully vaccinated children (Cohort 1):

- Included 806 children, aged less than 3.5 years.
- All children completed Health Center records of three doses of pentavalent vaccine and of OPV.
- Antibodies against tetanus was used as a proxy for the vaccination session attendance.
- In 2013 and 2014, samples were collected in Bolikhamxay and Vientiane provinces
- In 2015/2016, samples were collected in Khammouane province.
- The weight-for-height Z-scores (WHZ), height-for-age Zscores (HAZ) and weight-for-age Z-scores (WAZ) nutritional indicators were measured as nutritional indicators.
- Birthplace was recorded.

Children from remote areas (Cohort 2):

- Included 90 children aged less than 5 years were recorded in Xam Tai and Kuan from Huaphan province.

Children with unknown vaccination status (Cohort 3):

- Included 320 children aged less than 9 years
- In 2012, samples were measured from Bolikhamxay, Vientiane and Luang Prabang provinces.

Blood donors (Cohort 4):

- Included 528 blood donors, aged 16 to 56 years in 2014
- Unknown vaccination status from Vientiane, Huaphan, Khammouane, and Xaiyabury provinces.

Healthcare workers (Cohort 5):

- Included 700 people aged between 15 and 69 years in 2013
- Samples were collected in 3 central, 2 provincial and 8 district hospitals located in Vientiane capital, Huaphan and Bolikhamxay provinces respectively.

Similar to the simulations for the UCI datasets, the available datasets were randomly divided into training, validation and testing subsets. Out of 2448 samples, 1958, 244 and 244 sets were taken for the training, validation and testing of the WOA-RVFL and RVFL models. Each model is executed 25 independently runs. Table 4 shows the descriptive statistics of two major input parameters included in the model development namely Age and Titers. The other considered input parameters are the Cohort type which has five groups, Sex input which either male or female, and the Province which include nine places. The output parameter is Polio Immunoglobulin G (IgG) which includes three groups namely positive, equivocal, and negative.

**Table 4:** Descriptive statistics of the variables included in the VDPV outbreak model development.

	Age	Titers
Min	5	0.8933
Max	69	203.400
Average	25.67	54.472

**Figure 6:**
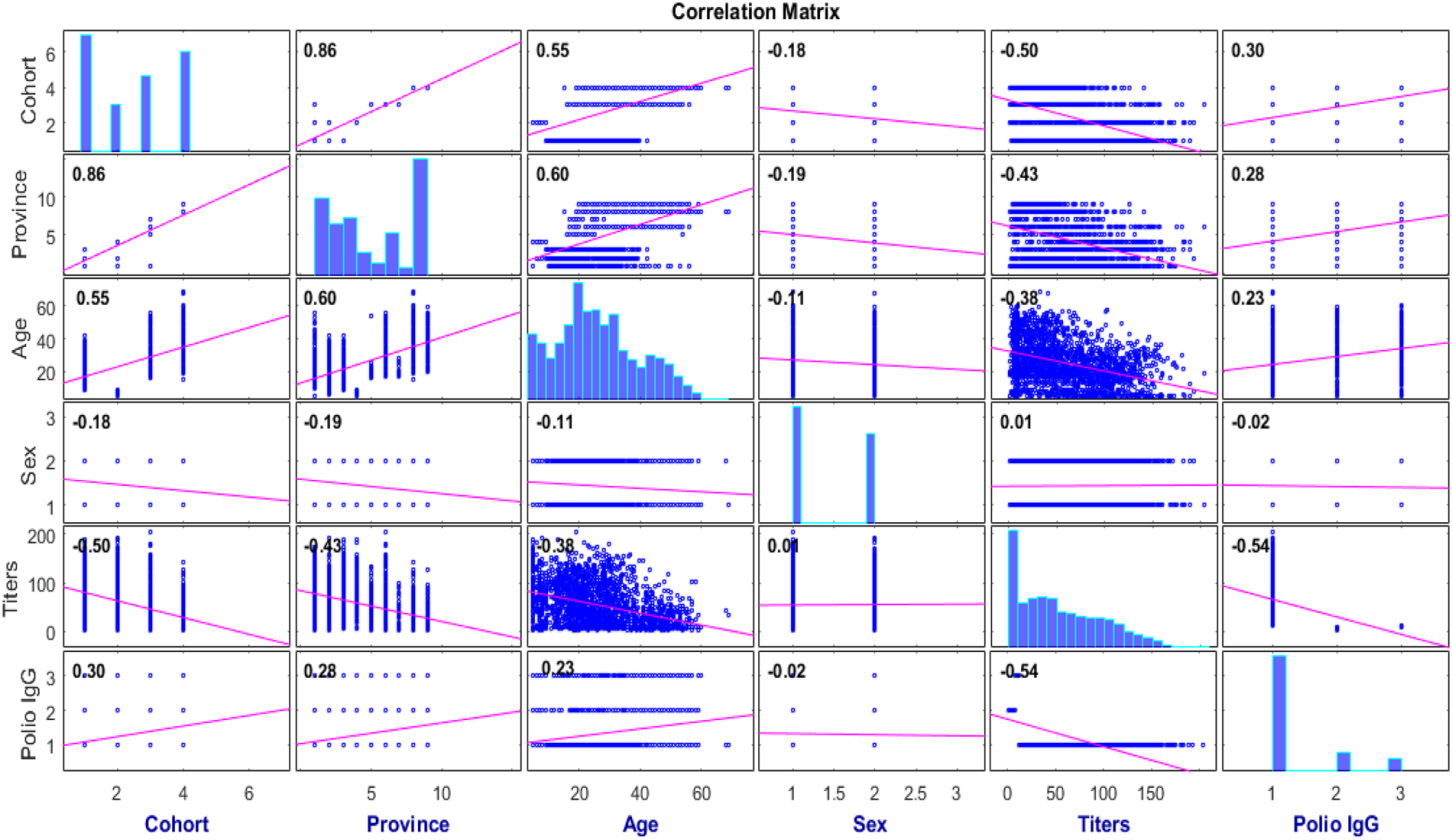
The correlation between the parameters included in the model development.

Fig. 6 depicts the correlation between the five parameters and with Polio IgG. As seen, the sex parameter has the smallest correlation with the other parameters. Additionally, the Cohort type, Titers, Age, and Province are correlated with Polio IgG with value greater than 0.20.

## Results and discussion

A comparison of the predictions made by the WOA-RVFL and classical RVFL methods is given in Table 5. On average, the CPU times for training the WOA-RVFL and RVFL algorithms were, respectively, 3.67 s and 8.62 s for the VDPV outbreak database. As seen in Table 5, the WOA-RVFL model significantly outperforms the RVFL model in terms of Accuracy, Precision and Recall rates. This involves the results for both the training, validation and testing data.

**Table 5:** Prediction performance of the WOA-RVFL and RVFL methods for classifying the VDPV outbreak incidences.

Method	Set	Pre	Rec	Acc
RVFL	Train	77.19	76.29	91.24
	Validation	72.38	75.93	92.78
	Test	74.90	71.45	89.36
WOA-RVFL	Train	100	100	100
	Validation	93.31	94.41	96.28
	Test	94.13	95.12	98.30

Moreover, the obtained results are in line with what was detected during the outbreak, where participants born before vaccination were significantly less to be seropositive. These results agree with the outbreak epidemiology. Antibodies neutralization against all poliovirus serotypes were diagnosed in all children. Likewise, antibodies neutralization against all serotypes was diagnosed in all health care workers. In addition, the WOA-RVFL method has figured out the IgG in the fully vaccinated 3.5 aged children class. In addition, the antibody seroprevalence of unvaccinated children, from marginalized areas, was found to be lower than vaccinated children. On the other hand, healthcare workers are classified to have a lower seroprevalence antibody than blood donors. Noteworthy, the proposed model categorizes both the children aged less than 1 year and younger adults to have antibodies more than older ages, supporting the idea that antibody levels were negatively associated with age.

However, VDPV outbreaks become ever-more interdisciplinary problem. In this context, scientists need to address how the revolutionary ML approaches can analyze the enormous amounts of data pouring in from epidemiology and immunology to sustain the clinical diagnostic tools [41]. The proposed WOA-RVFL approach presents an efficient methodological contribution to both ML and mathematical programming together with relevant insights into immunization evaluation. The WOA-RVFL analyzed the disparity between the different immunology assays. It is worth mentioning that high- risk countries may benefit from the proposed WOA-RVFL method for evaluating different immunization program. This can be particularly important for the cases that involve uncertain vaccination coverage or emergence virus neutralization tests (VNT).

In the polio-free areas considering seropositivity by ELISA, the proposed WOA-RVFL method can discriminate the trivalent vaccination from vulnerability to VDPV. Nonetheless, the improved ELISA must be serotype distinct, and negativity thresholds should be studied for the specificity and sensitivity. It should be noted that out of the five examined cohorts, both healthcare workers (cohort 5) and children (cohort 1) were analyzed by VNT^35^. The WOA-RVFL method handled the healthcare workers as a practical example of an adult with a high risk during the outbreak since they are at a higher risk for exposure to infections with a possibility to transfer the infection from a specific cohort to another. Thus, implementing healthcare worker in the proposed model helps understand the epidemiology of the outbreak to prevent the spread of disease from health care worker to patients, many of whom may be highly susceptible to infections and related complications. Therefore, it is important to track the immunization and vaccination in professionals and to ensure their ability to perform critical caring for patients.

The WOA-RVFL algorithm can observe the ELISA serologies of the other children and adult cohorts matched with results of groups that tested by VNT^35^. The WOA-RVFL suggests a high efficiency of outreach vaccination activities since the children from the remote area were equally well protected as the fully vaccinated children. The lower seropositivity rates were classified and predicted in fully vaccinated and unknown status children. This is compatible with the first clinical VDPV outbreak cases that occurred in the same area^36^. Given these features, the WOA-RVFL supports the idea of repairing the deficiencies associated with vaccine management that affect directly on vaccination efficacy.

### Phase III: Comparison with other meta-heuristic methods

In this section, the performance of the proposed WOA-RVFL is compared with meta-heuristic techniques which used to determine the optimal parameters of RVFL. These methods include particle swarm optimization (PSO), artificial bee colony (ABC), and sine-cosine algorithm (SCA). The parameter setting for each algorithm is given as the original paper, also, the common parameters such as the number of solutions, and the total number of iterations are set similar to the first experimental. In addition to, in this study, the dataset is divided into training and testing set using the 10-fold cross validation. This mean the dataset is split into 10 sets, one of them is used as testing and the other nine sets are used as training and this process is repeated 10 times until all sets are used as testing set.

Table 6 depicts the comparison results between the four algorithms using different measures. From this table it can be observed that the performance of the comparative algorithms has the same performance when the training set is used. Meanwhile, the accuracy of the WOA-RVFL, according to the testing sets, is better performance than other methods. Followed by the SCA-RVFL which allocated the second rank with nearly 97% and the performance of the ALO-RVFL is better than the MFO-RVFL. The same observation can be reached in terms of the precision and recall.

**Table 6:** The Comparison with other Meta-heuristic methods.

Method	Set	Pre	Rec	Acc	Method	Set	Pre	Rec	Acc
WOA-RVFL	Train	100	100	100	SCA-RVFL	Train	100	100	100
	Test	96.12	96.74	98.91		Test	95.16	94.45	97.25
ALO -RVFL	Train	100	100	100	MFO-RVFL	Train	100	100	100
	Test	92.83	93.24	95.23		Test	92.61	92.65	92.41

## Conclusions

This study presents a hybrid ML approach to predict the VDPV outbreak incidences. The proposed method called WOA-RVFL integrates the RVFL networks with the robust WOA optimization algorithm. It was shown that WOA notably improves the prediction accuracy of the RVFL network through finding suitable parameter configuration for this algorithm. The classification performance of the proposed WOA-RVFL method is first verified using a number of datasets from the UCI ML repository. The WOA-RVFL algorithm was deployed to track the VDPV outbreak incidences and Polio IgG recently occurred in several provinces in Lao. Based on the results, the WOA-RVFL algorithm is efficient in detecting the VDPV outbreak incidences and outperforms the traditional RVFL method. Future research can focus on implementing the WOA-RVFL algorithm to improve quantitative structure–activity relationship (QSAR) models and to other public health surveillance applications.
