# Author Correction: Prediction of the Vaccine-derived Poliovirus Outbreak Incidence: A Hybrid Machine Learning Approach

**DOI:** 10.1038/s41598-020-67204-1

**Published:** 2020-07-06

**Authors:** Ahmed A. Hemedan, Mohamed Abd Elaziz, Pengcheng Jiao, Amir H. Alavi, Mahmoud Bahgat, Marek Ostaszewski, Reinhard Schneider, Haneen A. Ghazy, Ahmed A. Ewees, Songfeng Lu

**Affiliations:** 1 Luxembourg Centre for Systems Biomedicine (LCSB), University of Luxembourg, EschsurAlzette, Luxembourg; 2 Department of Mathematics, Faculty of Science, Zagazig University, Zagazig, Egypt; 3 Ocean College, Zhejiang University, Zhoushan, 316021 Zhejiang China; 4 Department of Civil and Environmental Engineering, University of Pittsburgh, Pittsburgh, PA USA; 5 Department of Bioengineering, University of Pittsburgh, Pittsburgh, PA USA; 6 Research Group Immune- and Bio-markers for Infection, the Center of Excellence for Advanced Sciences, the National Research Center, Cairo, Egypt; 7 Therapeutic Chemistry Department, The National Research Center, Cairo, Egypt; 8 Biotechnology Department, Animal Health Research Institute, Kafrelsheikh, Egypt; 9 Department of Computer, Damietta University, Damietta El-Gadeeda City, Egypt; 10 School of Computer Science& Technology, Huazhong University of Science and Technology, Wuhan, 430074 China; 11 Department of Computer Science and Information Engineering, Asia University, Taichung, Taiwan

Correction to: *Scientific Reports* 10.1038/s41598-020-61853-y, published online 19 March 2020

In the original version of this Article, Amir H. Alavi was incorrectly affiliated with ‘Department of Civil and Environmental Engineering, University of Missouri, Columbia, MO, USA’ and ‘Department Biomedical, Biological and Chemical Engineering, University of Missouri, Columbia, MO, USA’. The correct affiliations are listed below.

Department of Civil and Environmental Engineering, University of Pittsburgh, Pittsburgh, PA, USA

and

Department of Computer Science and Information Engineering, Asia University, Taichung, Taiwan

and

Department of Bioengineering, University of Pittsburgh, Pittsburgh, PA, USA

This error has now been corrected in the PDF and HTML versions of the Article.

